# Severity of underweight affects the development of nontuberculous mycobacterial pulmonary disease; a nationwide longitudinal study

**DOI:** 10.1038/s41598-022-21511-x

**Published:** 2022-10-13

**Authors:** Ji Young Kang, Kyungdo Han, Mee Kyoung Kim

**Affiliations:** 1 Division of Pulmonology, Department of Internal Medicine, Cheju Halla General Hospital, Jeju, 63127 South Korea; 2 Department of Statistics and Actuarial Science, Soongsil University, Seoul, 06978 South Korea; 3 Division of Endocrinology and Metabolism, Department of Internal Medicine, Yeouido St. Mary’s Hospital, College of Medicine, The Catholic University of Korea, #10 63-ro, Yeongdeungpo-gu, Seoul, 07345 South Korea

**Keywords:** Medical research, Risk factors

## Abstract

Regarding to known association between underweight and non-tuberculous mycobacterial pulmonary disease (NTM-PD), the underweight was simply categorized as body mass index (BMI) less than 18.5 kg/m^2^, mainly because of its low prevalence. We aimed to better define the impact of BMI severity on NTM-PD development. We analysed health data from 4,332,529 individuals examined in 2009 and followed up until December 2017 to determine the incidence of NTM-PD. Based on the BMI in kg/m^2^, the population was categorized into mild (17.00–18.49), moderate (16.00–16.99), and severe underweight (< 16.00) groups. Using Cox proportional-hazards analyses, hazard ratios for NTM-PD were calculated according to the severity of underweight in reference to normal BMI (18.50–22.99). Over a median follow-up of 5.6 ± 1.2 years, 6223 participants developed NTM-PD. Risk of NTM-PD increased significantly with the severity of underweight: multivariate adjusted hazard ratios (95% confidence intervals) for mild, moderate, and severe underweight were 2.34 (2.17–2.52), 3.50 (3.07–3.99), and 4.35 (3.63–5.21), respectively. In subgroup analysis, being younger (< 65 years old) or male exacerbated the effect of severe underweight on the risk of NTM-PD. This study proved that as underweight categories became more severe, the risk of NTM-PD increased proportionally.

## Introduction

Being underweight is associated with many critical illnesses including respiratory diseases^1–5^_._ In some pulmonary infectious diseases, such as pneumonia or pulmonary tuberculosis, underweight is associated not only with the development of the diseases but also with the mortality^2,6–8^_._ In a recent meta-analysis, coronavirus disease 2019 (COVID-19) patients showed a J-shape relationship between body mass index (BMI) and mortality, suggesting that both underweight and obese COVID-19 patients had a higher mortality risk than patients with normal weight^9^_._ However, while overweight patients are already considered to be at risk of COVID-19 for assigning vaccine priority, underweight patients have not received comparable attention. Most previous clinical studies have simply classified individuals with a BMI less than 18.5 kg/m^2^ into a single group, mainly because of the low prevalence of underweight.

Non-tuberculous mycobacteria (NTM) are highly abundant in environmental niches such as soil and natural and drinking water sources, often leading to high rates of human–pathogen contact. The microbes commonly invade the human respiratory system and cause NTM pulmonary disease (NTM-PD). Recently, incidences of NTM-PD have increased substantially worldwide, because of advances in diagnostic techniques, increased clinical awareness of the disease, and a growing population vulnerable to NTM infection^10^. Previous reports have shown that low BMI increases the risk of NTM-PD and affects in-hospital or long-term mortality^11–14^.

However, it is not known whether severity of underweight is associated with a higher risk of NTM in a dose-responsive fashion. We aimed to investigate the incidence and risk of NTM-PD stratified by detailed underweight categories according to the World Health Organization (WHO) classification^15^, as mild (BMI 17.0–18.49), moderate (16.00–16.99), and severe underweight (< 16.00), using large-scale data sets from the Korean National Health Insurance Service (NHIS) database.

## Methods

### Data source and study population

**Figure 1:**
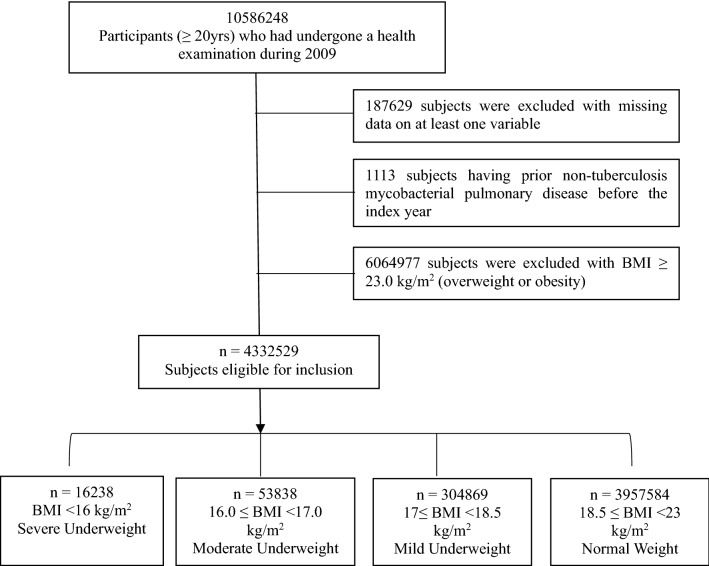
Flow chart of the study population.

The NHIS is a mandatory universal health insurance service managed by the South Korean government. The NHIS database covers virtually the entire Korean population and contains information on demographics and all medical services rendered (with diagnostic codes as per the International Statistical Classification of Diseases and Related Health Problems, 10th edition (ICD-10)), as well as all prescription medications dispensed. Enrollees in the National Health Insurance Corporation are recommended to undergo standardized medical examinations every 2 years. Reports from these health examination are also available from the NHIS database. In our study, we included the data from people (≥ 20 years) who underwent a health examination through the NHIS between 1 January 2009 and 31 December 2009 (n = 10,586,248). We excluded individuals with a prior diagnosis of NTM-PD before enrolment (n = 1113), those with any missing variables (n = 187,629), and those who were overweight or obese (n = 6,064,977). The remaining 4,332,529 eligible subjects were included in the analyses and followed up until the date of death or until 31 December 2017 (Fig. 1). All procedures related with human participants in this study were conducted in accordance with the ethical standards of the Helsinki Declaration. This study was approved by the Institutional Review Board of The Catholic University of Korea (IRB No: KC21ZISI0865). The requirement for written informed consent was waived by the review board because anonymized, de-identified information was used for analysis.

### Measurements and definitions

The general medical examination included history taking, blood pressure measurement, blood sampling, urinalysis, and chest X-ray results. During the health examination, height, weight, and waist circumference (WC) were measured, and BMI was calculated by dividing weight (kg) by height (m) squared. Subjects were categorized according to BMI using the WHO Western Pacific Region guideline strata of underweight (< 18.5 kg/m^2^) and normal weight (18.5–22.9 kg/m^2^)^16^. The underweight population was further categorized into mild (BMI 17.00–18.49), moderate (16.00–16.99), and severe underweight (< 16.00). Blood samples for measurement of serum glucose and lipid levels were drawn after an overnight fast. Information on current smoking, alcohol consumption (heavy alcohol consumption defined as ≥ 30 g/day) and regular exercise was obtained by questionnaire. Comorbidities that can influence the risk of NTM-PD were also defined using the following ICD-10 codes: asthma (J45–J46), bronchiectasis (J47), chronic obstructive pulmonary disease (COPD) (J41-44), tuberculosis sequelae (B90.9), pneumoconiosis (J60-J62), diabetes mellitus (DM) (E10–E14), chronic kidney disease (CKD) (N18.1–N18.5 and N18.9), gastroesophageal reflux disease (GERD) (K21.0), solid cancer (C00–C97), connective tissue disease (M05, M06, M32, M35, and M45), hematologic malignancy (C90, C910, C920, C921, C922, C924–926, C928, and C930), transplantation (Z940, Z944, Z941 and Z942), human immunodeficiency virus infection (HIV) and acquired immune deficiency syndrome (B20–B24).

### Primary outcome and follow-up

The primary outcome was the incidence of NTM-PD. NTM-PD was defined by claims made under the ICD-10 diagnosis code A31.0 (pulmonary mycobacterial infection). We identified individuals with NTM-PD who were newly registered with A31.0 after the index date and had at least two ambulatory visits or hospital admissions with the same diagnosis code in 1 year. The study population was followed from baseline to the date of the primary event in which a subject was newly diagnosed with NTM-PD, or until 31 December, 2017, whichever came first.

### Statistical analyses

Baseline characteristics are presented as the mean ± SD or n (%). Participants were classified into four groups according to BMI categories; normal weight, mild, moderate, and severe underweight. The incidence rate of primary outcome was calculated by dividing the number of incident cases by the total follow-up duration (person-years). The Cox proportional-hazards model was used to estimate hazard ratios (HRs) and 95% confidence interval (CI) values for study outcomes according to the categories of underweight. The proportional-hazards assumption was assessed using the Schoenfeld residuals test, with a logarithm of the cumulative hazard functions based on Kaplan–Meier estimates. Over time, there was no significant departure from proportionality in the hazards. A multivariable-adjusted proportional hazards model was applied: model 1 was adjusted for age and sex; model 2 was additionally adjusted for smoking, alcohol intake, and regular exercise; model 3 was further adjusted for confounding comorbidities such as respiratory diseases, solid cancer, hematologic malignancy, transplantation, AIDS, GERD, connective tissue diseases, and DM. Additionally, we performed stratified analysis according to WC to estimate the effect of abdominal fat on the risk of NTM-PD. The potential effect modification by age, sex, respiratory disease, DM, any malignancy, or connective tissue disease was evaluated through the stratified analysis and interaction testing using a likelihood ratio test. Statistical analyses were performed using SAS version 9.4 (SAS Institute Inc., Cary, NC, USA), and a P value < 0.05 was considered to be significant.

## Results

### Baseline characteristics

Overall, 4,332,529 subjects were included in the analysis. The mean age of the study population was 45.0 years, 46.3% were male. Individuals in the severe underweight group were older and less likely to exercise regularly, and had a higher prevalence of DM, CKD, GERD, any malignancy, COPD, asthma, and bronchiectasis than those in the mild underweight group (Table 1). Over a median follow-up of 5.6 ± 1.2 years, 6223 participants developed NTM-PD.

**Table 1:** Baseline characteristics of subjects according to the baseline body mass index.

	Total population	Normal weight (18.5 ≤ BMI < 23.0, kg/m^2^)	Mild underweight (17.0 ≤ BMI < 18.5, kg/m^2^)	Moderate underweight (16.0 ≤ BMI < 17.0, kg/m^2^)	Severe underweight (BMI < 16.0, kg/m^2^)
N	4,332,529	3,957,584 (91.3%)	304,869 (7.04%)	53,838 (1.24%)	16,238 (0.37%)
Age (years)	45.0 ± 14.8	45.4 ± 14.5	40.3 ± 16.3	42.0 ± 18.6	48.4 ± 21.3
Sex (male)	2,007,154 (46.3)	1,881,527 (47.54)	103,046 (33.8)	17,035 (31.64)	5546 (34.15)
Waist circumference (cm)	73.6 ± 6.7	74.3 ± 6.3	66.7 ± 5.6	64.6 ± 5.8	63.8 ± 7.0
**Smoking status**					
Non-smoker	2,793,390 (64.47)	2,530,053 (63.93)	213,686 (70.09)	38,343 (71.22)	11,308 (69.64)
Ex-smoker	468,530 (10.81)	442,510 (11.18)	21,217 (6.96)	3508 (6.52)	1295 (7.98)
Current smoker	1,070,609 (24.71)	985,021 (24.89)	69,966 (22.95)	11,987 (22.26)	3635 (22.39)
Alcohol intake	266,472 (6.15)	249,463 (6.3)	14,055 (4.61)	2241 (4.16)	713 (4.39)
Regular exercise	686,703 (15.9)	650,923 (16.5)	30,090 (9.9)	4462 (8.3)	1228 (7.6)
Income (lower 25%)	784,447 (18.1)	715,787 (18.1)	55,454 (18.2)	10,038 (18.6)	3168 (19.5)
Systolic BP (mmHg)	118.26 ± 14.47	118.68 ± 14.43	113.82 ± 13.96	113.36 ± 14.65	114.48 ± 16.22
Diastolic BP (mmHg)	73.74 ± 9.64	73.97 ± 9.63	71.34 ± 9.31	71.15 ± 9.5	71.83 ± 10.19
Fasting glucose (mg/dL)	93.8 ± 21.46	94.07 ± 21.48	90.84 ± 20.28	91.29 ± 22.7	93.7 ± 28.52
eGFR (mL/min/1.73 m^2^)	89.51 ± 45.09	89.18 ± 44.76	92.96 ± 48.42	93.25 ± 49.11	92.03 ± 43.44
Baseline TC (mg/dL)	187.61 ± 34.91	188.6 ± 35.0	177.8 ± 32.1	176.5 ± 32.4	177.9 ± 35.4
Diabetes mellitus	234,658 (5.42)	221,355 (5.59)	10,124 (3.32)	2168 (4.03)	1011 (6.23)
Chronic kidney disease	263,790 (6.09)	242,445 (6.13)	16,649 (5.46)	3361 (6.24)	1335 (8.22)
HIV/AIDS	525 (0.01)	486 (0.01)	34 (0.01)	2 (0)	3 (0.02)
GERD	690,261 (15.9)	634,270 (16.0)	44,855 (14.7)	8320 (15.5)	2816 (17.3)
**Connective tissue diseases**					
Rheumatoid arthritis	7185 (0.17)	6289 (0.16)	668 (0.22)	164 (0.3)	64 (0.39)
Systemic lupus erythematosus	1614 (0.04)	1382 (0.03)	185 (0.06)	37 (0.07)	10 (0.06)
Systemic sclerosis	269 (0.01)	230 (0.01)	28 (0.01)	4 (0.01)	7 (0.04)
Ankylosing spondylitis	1887 (0.04)	1696 (0.04)	150 (0.05)	32 (0.06)	9 (0.06)
Any malignancy (solid cancer)	45,789 (1.06)	41,517 (1.05)	3274 (1.07)	715 (1.33)	283 (1.74)
Hematologic malignancy	546 (0.01)	514 (0.01)	26 (0.01)	4 (0.01)	2 (0.01)
**Transplantation status**					
Kidney	963 (0.02)	851 (0.02)	92 (0.03)	16 (0.03)	4 (0.02)
Liver	350 (0.01)	328 (0.01)	18 (0.01)	3 (0.01)	1 (0.01)
**Respiratory diseases**					
COPD	221,662 (5.12)	199,318 (5.04)	16,701 (5.48)	3826 (7.11)	1817 (11.19)
Asthma	294,143 (6.79)	267,691 (6.76)	20,527 (6.73)	4254 (7.9)	1671 (10.29)
Bronchiectasis	17,890 (0.41)	15,596 (0.39)	1591 (0.52)	446 (0.83)	257 (1.58)
TB sequelae	6935 (0.16)	5742 (0.15)	770 (0.25)	256 (0.48)	167 (1.03)
Pneumoconiosis	710 (0.02)	623 (0.02)	53 (0.02)	25 (0.05)	9 (0.06)

Data are expressed as the means ± SD, median (25–75%), or n (%).

*BP* blood pressure, *eGFR* estimated glomerular filtration rate, *HDL* high-density lipoprotein, *LDL* low-density lipoprotein, *TC* total cholesterol, *TG* triglyceride, *HIV* human immunodeficiency virus infection, *AIDS* acquired immune deficiency syndrome, *GERD* gastroesophageal reflux disease, *COPD* chronic obstructive pulmonary disease.

### Risk of NTM-PD according to underweight categories

The severity of underweight was significantly associated with the risk of NTM-PD. This association becomes more evident after adjusting for demographic factors (model 1 and 2; Table 2) and non-communicable disease-related factors (model 3; Table 2). There was a significant graded increase in risk of NTM-PD with the severity of underweight. Multivariate adjusted hazard ratios (HRs) (95% confidence interval [CI]) for mild, moderate, and severe underweight were 2.34 (2.17–2.52), 3.50 (3.07–3.99), and 4.35 (3.63–5.21), respectively.

**Table 2:** Risk of non-tuberculosis mycobacterial pulmonary disease according to the severity of underweight.

	N	Events (n)	Incidence rate^a^	Model 1 (HRs, 95% CI)	Model 2 (HRs, 95% CI)	Model 3 (HRs, 95% CI)
**Body mass index**						
< 16.0 kg/m^2^	16,238	122	0.842	5.22 (4.36, 6.25)	5.37 (4.48, 6.43)	4.35 (3.63, 5.21)
16.0–16.99 kg/m^2^	53,838	237	0.454	3.83 (3.36, 4.36)	3.95 (3.47, 4.50)	3.50 (3.07, 3.99)
17.0–18.49 kg/m^2^	304,869	775	0.255	2.47 (2.29, 2.66)	2.53 (2.34, 2.73)	2.34 (2.17, 2.52)
18.5–22.99 kg/m^2^	3,957,584	5089	0.127	1 (ref.)	1 (ref.)	1 (ref.)
*P* for trend^b^				< 0.0001	< 0.0001	< 0.0001

Model 1, adjusted for age and sex; Model 2, model 1 + smoking, alcohol intake, and regular exercise; Model 3, model 2 + respiratory diseases, solid cancer, hematologic malignancy, transplantation, AIDS, GERD, connective tissue diseases, diabetes mellitus.

^a^Per 1000 person-years.

*HRs* hazards ratios, *95% CI* 95% confidence interval.

^b^*P*-values for the trend were < 0.0001 for all variables because of the large size of the study population.

**Figure 2:**
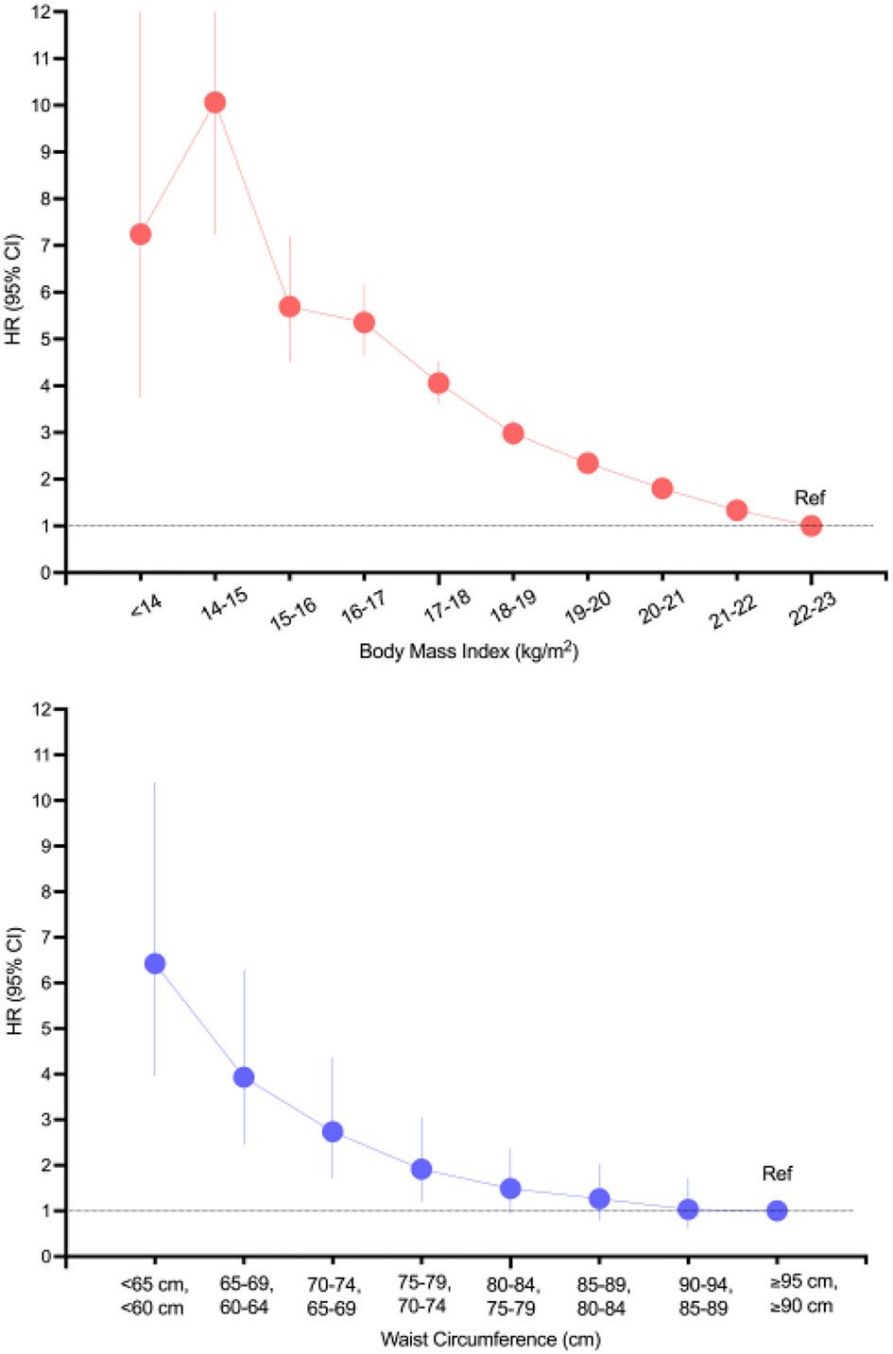
Hazard ratios (HRs) and 95% confidence intervals of non-tuberculous mycobacterial pulmonary disease according to body mass index (BMI) or waist circumference (WC). The group with baseline BMI 22–23 kg/m^2^ or WC ≥ 95 cm in men/ ≥ 90 cm in women was considered the reference group. Adjusted for age, sex, smoking, alcohol drinking, regular exercise, respiratory diseases, solid cancer, hematologic malignancy, transplantation, AIDS, GERD, connective tissue disease, DM.

We investigated the risk of NTM-PD for each decrease in BMI by 1 kg/m^2^ or each decrease in WC by 5 cm (Fig. 2). The reference group was defined as subjects with BMI 22–22.9 kg/m^2^. We found that the risk of NTM-PD increased linearly for every 1 kg/m^2^ decrease in BMI. For example, subjects with a BMI of 15–15.9 kg/m^2^ had a nearly 5.6 times higher risk of NTM-PD than those with a reference BMI of 22–22.9 kg/m^2^. Subjects with a BMI of 14–14.9 kg/m^2^ had an almost 10 times higher risk of NTM-PD than those with a reference BMI of 22–22.9 kg/m^2^. As for WC, the reference group was defined as subjects with WC ≥ 95 cm in men or ≥ 90 cm in women. The risk of NTM-PD increased from WC < 80 cm in men or < 75 cm in women. For example, the HR (95% CI) of NTM-PD was 1.92 (1.20–3.05) in subjects with WC 75–79 cm in men or WC 70–74 cm in women. The HR (95% CI) of NTM-PD was 6.42 (3.97–10.39) in subjects with WC < 65 cm in men or < 60 cm in women.

### Risk of NTM-PD according to waist circumferences and severity of underweight

Subjects were classified based on WC into three sex-specific tertiles; tertile1 (< 75 cm in men, < 68 cm in women), tertile2 (75–79 cm in men, 68–72 cm in women), and tertile3 (≥ 80 cm in men, ≥ 73 cm in women) (Table 3). The group with normal weight and the highest tertile (T3) of WC was considered the reference group. In the stratification analysis by WC, the severity of underweight was associated with a higher risk of NTM-PD in all tertiles. The highest risk for NTM-PD was observed in the severe underweight and the lowest WC (HR 6.26, 95% CI 5.17–7.58).

**Table 3:** Risk of non-tuberculosis mycobacterial pulmonary disease according to waist circumferences (WC) and severity of underweight.

Body mass index	Waist circumstance	N	Events (n)	Incidence rate^a^	Model 1 (HRs, 95% CI)	Model 2 (HRs, 95% CI)	Model 3 (HRs, 95% CI)
< 16.0 kg/m^2^	T1: < 75 cm in men, < 68 cm in women	13,920	112	0.89	7.64 (6.31, 9.24)	7.83 (6.47, 9.47)	6.26 (5.17, 7.58)
	T2: 75–79 cm in men, 68–72 cm in women	1220	7	0.72	2.95 (1.40, 6.19)	3.07 (1.46, 6.44)	2.38 (1.13, 5.01)
	T3: ≥ 80 cm in men, ≥ 73 cm in women	1098	3	0.35	1.16 (0.38, 3.61)	1.20 (0.39, 3.72)	1.13 (0.36, 3.50)
16.0–16.99 kg/m^2^	T1: < 75 cm in men, < 68 cm in women	46,807	205	0.45	5.31 (4.60, 6.13)	5.42 (4.69, 6.25)	4.73 (4.09, 5.46)
	T2: 75–79 cm in men, 68–72 cm in women	4804	22	0.50	3.09 (2.03, 4.70)	3.29 (2.16, 5.01)	3.07 (2.02, 4.67)
	T3: ≥ 80 cm in men, ≥ 73 cm in women	2227	10	0.54	1.98 (1.06, 3.68)	2.08 (1.12, 3.87)	1.99 (1.07, 3.71)
17.0–18.49 kg/m^2^	T1: < 75 cm in men, < 68 cm in women	239,705	591	0.25	3.46 (3.16, 3.79)	3.50 (3.19, 3.84)	3.27 (2.98, 3.59)
	T2: 75–79 cm in men, 68–72 cm in women	49,062	135	0.28	2.51 (2.11, 2.99)	2.60 (2.18, 3.09)	2.35 (1.97, 2.80)
	T3: ≥ 80 cm in men, ≥ 73 cm in women	16,102	49	0.33	1.71 (1.29, 2.27)	1.78 (1.34, 2.37)	1.64 (1.23, 2.17)
18.5–22.99 kg/m^2^	T1: < 75 cm in men, < 68 cm in women	1,049,852	1324	0.12	1.78 (1.66, 1.91)	1.76 (1.63, 1.89)	1.75 (1.62, 1.87)
	T2: 75–79 cm in men, 68–72 cm in women	1,421,858	1701	0.12	1.32 (1.24, 1.41)	1.31 (1.23, 1.40)	1.31 (1.23, 1.40)
	T3: ≥ 80 cm in men, ≥ 73 cm in women	1,485,874	2064	0.14	1 (ref.)	1 (ref.)	1 (ref.)
*P* for trend^b^					< 0.0001	< 0.0001	< 0.0001

Model 1, adjusted for age and sex; Model 2, model 1+ smoking, alcohol intake, and regular exercise; Model 3, model 2+ respiratory diseases, solid cancer, hematologic malignancy, transplantation, AIDS, GERD, connective tissue diseases, diabetes mellitus.

*HRs* hazards ratios, *95% CI* 95% confidence interval.

^a^Per 1000 person-years.

^b^*P*-values for the trend were < 0.0001 for all variables because of the large size of the study population.

### Subgroup analysis

When we performed stratified analysis according to age (< 65 years or ≥ 65 years), sex, and presence of respiratory disease, DM, any malignancy, or connective tissue disease, age and sex were found to be effect modifiers. Being in a younger age group or male exacerbated the effect of severe underweight on the risk of NTM-PD (Table 4).

**Table 4:** Risk of non-tuberculosis mycobacterial pulmonary disease in subgroups according to age, sex, presence of diabetes mellitus, respiratory disease, any malignancy, and connective tissue diseases.

	Subgroup		Severity of underweight			*P* for interaction
		Normal weight	Mild (HRs, 95% CI)	Moderate (HRs, 95% CI)	Severe (HRs, 95% CI)	
Age	< 65	1(ref.)	2.51 (2.27, 2.76)	4.23 (3.56, 5.03)	5.55 (4.28, 7.20)	0.013
	≥ 65	1 (ref.)	2.89 (2.56, 3.26)	3.92 (3.20, 4.80)	5.41 (4.19, 6.98)	
Sex	Men	1 (ref.)	2.80 (2.51, 3.12)	4.76 (3.99, 5.68)	5.41 (4.18, 7.00)	< 0.001
	Women	1 (ref.)	1.93 (1.74, 2.15)	2.54 (2.09, 3.09)	3.58 (2.77, 4.62)	
Respiratory diseases	No	1 (ref.)	2.38 (2.17, 2.61)	3.73 (3.16, 4.40)	4.80 (3.77, 6.11)	0.308
	Yes	1 (ref.)	2.32 (2.04, 2.64)	3.21(2.59, 3.97)	4.01 (3.04, 5.27)	
Diabetes mellitus	No	1 (ref.)	2.35 (2.17, 2.54)	3.50 (3.06, 4.01)	4.37 (3.62, 5.26)	0.974
	Yes	1 (ref.)	2.30 (1.64, 3.21)	3.48 (1.96, 6.21)	4.23 (1.99, 9.02)	
Any malignancy	No	1 (ref.)	2.36 (2.19, 2.55)	3.46 (3.03, 3.96)	4.48 (3.73, 5.37)	0.185
	Yes	1 (ref.)	1.83 (1.17, 2.87)	4.57 (2.53, 8.26)	1.10 (0.15, 7.93)	
Connective tissue diseases	No	1 (ref.)	2.33 (2.16, 2.52)	3.53 (3.10, 4.03)	4.21 (3.50, 5.07)	0.088
	Yes	1 (ref.)	3.20 (1.77, 5.79)	1.74 (0.42, 7.26)	12.63 (4.90, 32.55)	

Adjusted for age, sex, smoking, alcohol intake, regular exercise, respiratory diseases, solid cancer, hematologic malignancy, transplantation, AIDS, GERD, connective tissue diseases, diabetes mellitus.

*HR* hazards ratios, *95% CI* 95% confidence interval.

## Discussion

In this study, we classified underweight into three subgroups—mild, moderate, and severe—according to WHO classification and, and for the first time investigated the association between the the severity of underweight and the risk of developing NTM-PD. We confirmed that the greater the degree of severe underweight, the higher risk of NTM-PD. Subjects with severe underweight (BMI < 16 kg/m^2^) had a 4.4 times higher risk of NTM-PD than those of normal weight. This finding is strongly consistent after adjusting various parameters related to the development of NTM-PD-demographics, smoking, underlying diseases such as GERD, malignancy, or chronic respiratory diseases. Intriguingly, even under the normal weight category, a 1 unit decrease in BMI increased the risk of developing NTM-PD, suggesting an inverse correlation.

Some epidemiologic studies^14,17^ have shown a higher prevalence of NTM-PD in slender postmenopausal women. In our study, subjects with severe underweight were older and included more women compared with the normal weight group. Slender, older women appear to be more susceptible to NTM-PD, which might be because underweight is more prevalent in women^18^. In recent times, underweight in women has been one of the most prominent public health issues in Asia^19^.

We analyzed a further stratification by WC, a representative parameter of abdominal visceral fat, in the study group classified by BMI. Visceral fat is considered as more strongly relevant to clinical diseases such as diabetes, metabolic syndrome, or cardiovascular disease, than BMI. To measure excessive body fat accurately, instruments such as dual-energy X-ray absorptiometry, bioimpedance analysis (BIA), computed tomography (CT), or magnetic resonance imaging can be used. However, such methods are not easily accessible, especially in large-scale epidemiologic studies. The group with severe underweight and the lowest WC had the highest risk of NTM-PD, showing adjusted HR of 6.26 (95% CI 5.17–7.58). This association with WC was also observed within the same underweight category, suggesting that abdominal fat may have a protective effect on the risk of developing NTM-PD. It was recently reported that low fat mass was associated with the risk of NTM in patients with or without bronchiectasis^14,20,21^. As for other infectious diseases, such as tuberculosis or HIV infection, the protective effect of body fat was confirmed^22^. One hypothesis for this association between low body fat and NTM-PD involves altered adipokine secretion by adipose tissue. During underweight, serum leptin, which is involved in lymphopoiesis and production of protective cytokines, is decreased, while adiponectin, which has anti-inflammatory function, is increased, collectively inducing susceptibility to NTM infection^14,23^.

The current study has several strengths. We included a sufficient number of subjects with underweight and found that the lower the BMI, the higher risk of NTM-PD, without a lower limit of BMI. Moreover, this study was a large-scale longitudinal study in the general population and not targeted at specific disease groups such as patients with other lung diseases, so the result could be applied to all population. In our study, the incidence rate of NTM-PD in the population with normal weight was 12.7 per 100,000 person-years, similar to that found in the previous study^24^. Comparing with normal weight, any category of underweight imposed a significantly higher risk of NTM-PD. The incidence rate in subjects with mild underweight was 25.5 per 100,000 person-years. Finally, we used data on WC to assess the effect of abdominal fat mass on the association of underweight and NTM-PD.

By contrast, there are some weaknesses to be addressed. First, as it is an observational study, we are not able to conclude clearly that there is a clear cause- and-effect relationship between underweight and NTM-PD. Second, although we attempted to control possible confounders statistically, there is a possibility of residual confounders. Third, we could not evaluate lean and fat mass through BIA or CT, or measure cytokines or hormone associated with underweight. A large-scale, prospective cohort study is required to confirm the relationship between underweight and NTM-PD and to verify the underlying mechanisms.

In conclusion, this study revealed that severity of underweight, not just being underweight, was significantly associated with the risk of NTM-PD, and high WC in the same underweight group could have protective role for the disease. In real practice, assessment of nutritional status and its support during underweight in a subject might be one of the helpful strategies for preventing the development of NTM-PD.

## Data Availability

The dataset analyzed in the present study was available from the corresponding author upon reasonable request.
